# Prediction of functional loss in emergency surgery is possible with a simple frailty screening tool

**DOI:** 10.1186/s13017-021-00356-1

**Published:** 2021-03-18

**Authors:** Davide Zattoni, Isacco Montroni, Nicole Marie Saur, Anna Garutti, Maria Letizia Bacchi Reggiani, Federico Ghignone, Giovanni Taffurelli, Giampaolo Ugolini

**Affiliations:** 1 Department of General Surgery, Policlinico S. Orsola-Malpighi, 40138 Bologna, Italy; 2 Department of General Surgery, Ospedale per gli Infermi, Viale Stadone, 9, –48018 Faenza, Italy; 3 Department of Surgery, Division of Colon and Rectal Surgery, University of Pennsylvania, 800 Walnut Street 20th floor, Philadelphia, PA USA; 4 Department of Geriatrics, Policlinico S. Orsola-Malpighi, 40138 Bologna, Italy; 5 Department of Cardiology–Statistics division, Policlinico S. Orsola-Malpighi, 40138 Bologna, Italy

**Keywords:** Functional outcome, Functional decline, Frailty assessment, Flemish version of Triage Risk Screening Tool, Emergency surgery

## Abstract

**Background:**

Senior adults fear postoperative loss of independence the most, and this might represent an additional burden for families and society. The number of geriatric patients admitted to the emergency room requiring an urgent surgical treatment is rising, and the presence of frailty is the main risk factor for postoperative morbidity and functional decline. Frailty assessment in the busy emergency setting is challenging. The aim of this study is to verify the effectiveness of a very simple five-item frailty screening tool, the Flemish version of the Triage Risk Screening Tool (fTRST), in predicting functional loss after emergency surgery among senior adults who were found to be independent before surgery.

**Methods:**

All consecutive individuals aged 70 years and older who were independent (activity of daily living (ADL) score ≥5) and were admitted to the emergency surgery unit with an urgent need for abdominal surgery between December 2015 and May 2016 were prospectively included in the study. On admission, individuals were screened using the fTRST and additional metrics such as the age-adjusted Charlson Comorbidity Index (CACI) and the ASA score. Thirty- and 90-day complications and postoperative decline in the ADL score where recorded. Regression analysis was performed to identify preoperative predictors of functional loss.

**Results:**

Seventy-eight patients entered the study. Thirty-day mortality rate was 12.8% (10/78), and the 90-day overall mortality was 15.4% (12/78). One in every four patients (17/68) experienced a significant functional loss at 30-day follow-up. At 90-day follow-up, only 3/17 patients recovered, 2 patients died, and 12 remained permanently dependent. On the regression analysis, a statistically significant correlation with functional loss was found for fTRST, CACI, and age≥85 years old both at 30 and 90 days after surgery. fTRST≥2 showed the highest effectiveness in predicting functional loss at 90 days with AUC 72 and OR 6.93 (95% CI 1.71–28.05). The institutionalization rate with the need to discharge patients to a healthcare facility was 7.6% (5/66); all of them had a fTRST≥2.

**Conclusion:**

fTRST is an easy and effective tool to predict the risk of a postoperative functional decline and nursing home admission in the emergency setting.

## Background

An increased number of senior adults are admitted to the emergency department requiring urgent or emergent surgical care [1, 2]. This population experiences poor surgical outcomes with high rates of postoperative complications, mortality, resource use, and a greater chance of being dependent at hospital discharge [3, 4]. Emergency surgery can also promote functional decline secondary to preoperative deconditioning and/or postoperative complications [5]. Losing preoperative abilities such as mental capacity, continence, mobility, or independence in daily activities is the most-feared postoperative event among geriatric patients [6] and could lead to sudden requirements for the assistance of a caregiver or discharge to a nursing facility [7]. Loss of independence impacts a patient’s quality of life and represents an economic and social burden for families and society [4].

The most important risk factor for functional decline is frailty [4, 8, 9]. The surgeon has few instruments to predict a postoperative functional loss in the busy emergency setting where a frailty assessment in not available or reliable [2, 7, 10]. A short frailty screening tool validated in a previous study, the Flemish version of the Triage Risk Screening Tool (fTRST), is effective in predicting 30- and 90-day morbidity and mortality after emergency abdominal surgery among older patients (≥70 years) [10].

The aim of this study is to analyze if the fTRST can also predict loss of independence in a group of previously independent senior adults undergoing emergency abdominal surgery.

## Methods

### Study population

Between December 2015 and May 2016, patients 70 years old and older were prospectively enrolled before undergoing emergency abdominal surgery at a tertiary hospital. Patients with a preoperative ADL score of 6/6 or 5/6 at the preoperative baseline were considered functionally independent and included in the study. The study was conducted under the Institutional Review Board (214/2016/O/OssN). Informed consent was obtained from every patient, and a health care proxy was used in cases of dementia or altered mental status.

### Study protocol

On admission, demographic data, activities of daily living (ADL), walking ability, Charlson Comorbidity Age Adjusted (CACI) score, the fTRST, and the American Society of Anesthesiology (ASA) score were recorded in a dedicated database.

The study-specific baseline assessment was performed by a senior general surgery resident (DZ) together with the resident on call who performed the standard admission history and physical exam, upon patient admission to the emergency surgery unit (ESU). In the case of emergent surgery, or if patients were incoherent, baseline assessment was obtained in the immediate postoperative period via interview of a family member.

Type and duration of surgery and postoperative morbidity and mortality were also recorded (DZ). Outcomes were re-assessed at 30 and 90 days after surgery. Follow-up consisted of an outpatient visit or a phone encounter depending on the patient’s status (DZ). Caregivers were interviewed in cases where patients were unable to answer.

### Functional loss risk factors

Preoperative frailty screening tools have been well established in the literature [10–16]. Thresholds have been reported as follows: fTRST score ≥ 2, ASA score ≥ 4, CACI ≥ 6, major surgery, and age ≥ 85 years old. The fTRST (Table 1) is based on five domains: presence of cognitive decline (2 points), living alone or caregiver not available or able (1 point), reduced mobility (necessity of a cane/walker or a caregiver’s aid) or falls in the past 6 months (1 point), hospitalized in the past 3 months (1 point), and polypharmacy (the cutoff for polypharmacy is established for this test when the patient takes ≥ 5 different medications) (1 point). The fTRST cutoff was selected based on previous analysis [10], and it is in line with the cutoff chosen by Kenis et al. in the medical oncology setting [17]. Elevated CACI score was considered a predictor of morbidity and mortality [12, 13]. Based on past works [10, 12], the cutoff was established as ≥ 6. “Major surgery” was defined as a surgical intervention involving ≥1 bowel resection or at least one anastomosis, gastric resection, splenectomy, or surgery performed for diffuse peritonitis. Bowel surgery as adhesiolysis or stoma creation without resection, appendectomies, laparoscopic cholecystectomies (without diffuse peritonitis), or hernia repair are considered intermediate-minor procedures. ASA score ≥ 4 has been described as a significant risk factor for morbidity and mortality in the geriatric population undergoing emergency procedure [18, 19]. As age is often a surrogate for frailty and since age has been included as a variable in past studies, it was included in this analysis, and people 85 and older were also considered at higher risk for postoperative complications [20].

**Table 1:** The Flemish version of the Triage Risk Screening Tool

Items of “The Flemish version of the Triage Risk Screening Tool”	Score	
	Yes	No
1. Presence of cognitive impairment (disorientation, diagnosis of dementia, or delirium)	2	0
2. Lives alone or no caregiver available, willing, or able	1	0
3. Difficulty with walking or transfers or fall(s) in the past 6 months	1	0
4. Hospitalized in the last 3 months	1	0
5. Polypharmacy: ≥ 5 medications	1	0

### Outcome measures

Patients were considered to be independent on the activities of daily living if ADL score was 6/6 or 5/6 on admission. A functional loss was established when a postoperative decline on ADL was observed 30 and 90 days after surgery from ADL 5-6 to ADL≤4.

All complications occurring during the hospital stay or within 90 days from discharge were recorded according to the Clavien-Dindo (CD) classification. CD ≥3 was considered a major complication. Postoperative outcomes were recorded during the hospital stay by a senior resident (DZ). The 30- and 90-day follow-up was assessed via office visit or phone encounter with patients and/or caregivers.

Patients were considered to be institutionalized when a permanent transfer to a nursing facility occurred for patients previously living at home.

### Statistical analysis

Statistical analyses were performed using Stata/SE 14.1 for Windows; continuous variables were expressed as mean ± standard deviation or median and interquartile range; categorical data were expressed as numbers (percentages). For group comparisons of categorical and continuous variables, chi-square test, Fisher’s exact test, Student’s *t*-test, or Mann-Whitney test was used, as appropriate.

Logistic regression analyses were performed in order to evaluate pre- and intraoperative variables as risk factors (or predictors) of 30-day and 90-day “significant” functional loss (∆≥2 points on ADL score). Included risk variables were fTRST score ≥ 2, ASA score ≥ 4, CACI ≥ 6, major surgery intervention, and age ≥ 85 years old.

The model discrimination and calibration were reported together with AIC (Akaike information criterion) and BIC (Bayesian information criterion) measures for comparing maximum likelihood models. Model discrimination was assessed calculating the area under the receiver operating characteristic (ROC) curve (AUC), whereas model calibration has been determined by Hosmer-Lemeshow (H-L) technique. All *p* values refer to two-tailed tests of significance. *p*<0.05 was considered significant. Given two models fit on the same data, the model with the smaller value of the information criterion is considered to be better.

## Results

### Patient characteristics

**Fig. 1:**
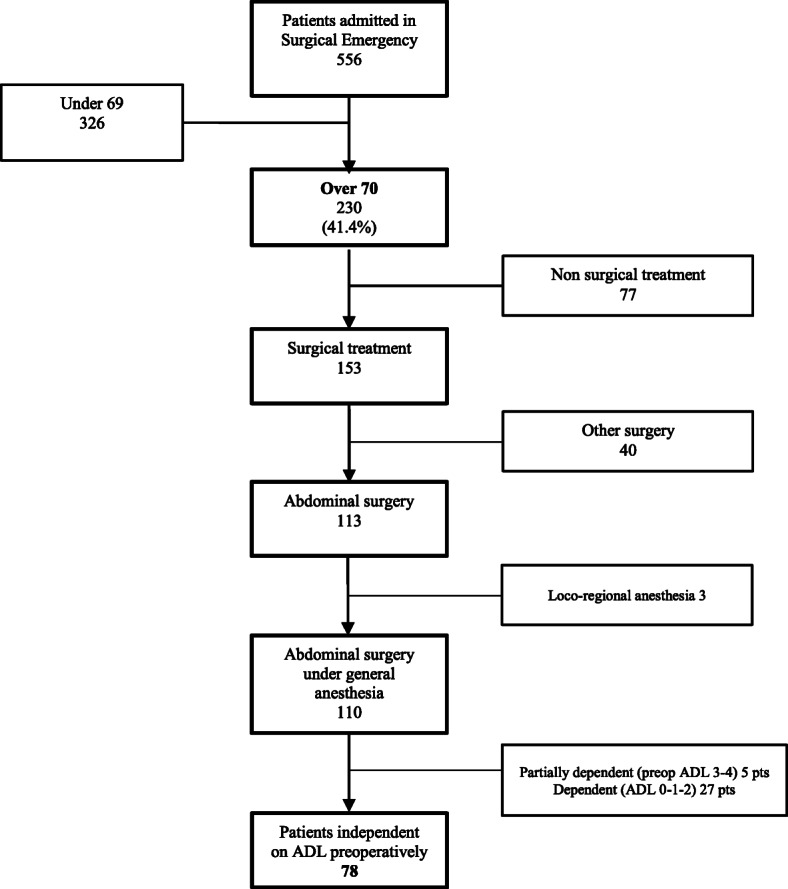
Population diagram

Between December 2015 and May 2016, 110 consecutive patients 70 years old and older underwent an abdominal urgent intervention under general anesthesia. Population diagram is reported on Fig. 1. Seventy-eight independent individuals on ADL (78/110; 70.9%) were included in the analysis.

No eligible patient refused to participate in the study or was lost at follow-up. Twenty-seven percent of the population included in the study analysis was ≥85 years of age or older (16 patients 70–74 years, 25 patients 75–79 years, 16 patients 80–84 years, 15 patients 85–89 years, 6 patients ≥ 90 years old). Median age was 78 years (range 70–96). Most of the patients came to the emergency room from home (65.4%), 26 patients were transferred from other units (33.3%), and only 1 patient lived in a nursing facility before admission.

Demographic data and patients’ conditions on admission are included in Table 2. Among patients with ADL ≥6, 85% had a score of 6/6 while 12 patients had a score of 5 (15%) being dependent on bathing or reporting urinary/fecal incontinence. The fTRST score was found to be ≥ 2 in 51.3% of the patients (40/78). The most frequent reported issues were polypharmacy (43/78 patients; 55.1%) and walking disability (31/78; 39.7%). One in four independent patients lived alone with no available caregiver. The most commonly represented ASA score was ASA 3 (68%). Almost half of the study population (38/78) presented a high comorbidity rate with elevated CACI score (CACI≥6).

**Table 2:** Demographic data

**Demographic data**	Age	Median (range)	78 (70–96)
		Over 85 n (%)	21 (27%)
	Gender	Male	34
		Female	44 (56.4%)
	ASA	I	0
		II	12 (15.4%)
		III	53 (67.9%)
		IV	12 (15.4%)
		V	1 (1.3%)
	CACI	Average (range)	5 (3–14)
		Score ≥6 (%)	38 (48.7%)
	fTRST	Score ≥2 (%)	40 (51.3%)
	Walking capacity	Any problem	54 (69.2%)
		With a cane	18 (23.1%)
		With two canes	2 (2.6%)
		With a walker	4 (5.1%)
	ADL	ADL 5 (%)	12 (15.4%)
		ADL 6 (%)	66 (84.6%)
**Diagnosis**	Bowel	Obstruction (cancer)	32 (11)
		Perforation (cancer)	6 (5)
		Diverticulitis	5
		Ischemia	4
		Bleeding (cancer)	4 (4)
	Cholecystitis	*n* (with peritonitis)	10 (3)
	Gastric/duodenal perforation	*n*	3
	Splenic injury	*n*	1
	Abdominal wall hernia	*n*	9
	Appendicitis	*n*	4
	**TOTAL**	***n*** (cancer %)	**78 (25.6%)**

### Surgical intervention

The details of the surgical procedures are shown in Table 3. Most of the patients underwent operations for large or small bowel diseases (51/78 patients), with twenty patients found to be affected by colorectal cancer (Table 3). A major intervention was performed in 41 cases (52.6%) including a colonic, small bowel, or gastric resection. Five cases of cholecystectomy were considered as major procedure, in four cases because of the presence of a diffuse biliary peritonitis requiring open approach or conversion to open procedure, and in one case because of the presence of duct obstruction requiring an endoscopic retrograde cholangiopancreatography during the same procedure. Thirty-eight procedures were performed of intermediate and minor surgery (cholecystectomies, appendectomies, hernia repair, adhesiolysis, stoma creation without bowel resection). Surgery was performed laparoscopically in 22% of the cases.

**Table 3:** Surgical intervention

Intervention		Procedures	Major procedures
Colonic resection	Right 6, left 5, partial 2, ileocecal 1, sigmoid 1, Hartmann 6, total colectomy 2	23 (4 lap)	23
Small bowel resection		8	8
Small bowel surgery (without resection)	adhesiolysis 16, stoma 2	18 (2 lap)	/
Cholecystectomy		10 (6 lap)	5
Appendectomy		4 (4 lap)	/
Hernia repair		9	/
Peptic ulcer repair		3 (1 lap)	2
Gastric resection		1	1
Splenectomy		1	1
Abdominal lavage		1 (1 lap)	/
**Total**		**78 (17 lap**—21.8%)	40 (51.3%)

*Lap=* laparoscopic

### Mortality

The postoperative 30-day mortality rate was 12.8% (10/78) and occurred shortly after the intervention in 9 cases out of 10. The most frequent cause of death was septic shock. Seven patients had ASA score ≥4 (7/10). Eight deaths were observed after a major procedure. The overall 90-day mortality rate was 15.4% (12/78). The two deaths that occurred between 31 and 90 days were secondary to progression of an end-stage neoplasia.

### Complications

Twenty patients (25.6%) experienced an uneventful postoperative course. Twenty-six patients (26/78, 33.3%) were transferred on ICU after the surgical procedure for postoperative monitoring.

The overall postoperative 30-day complication rate was 70.6% (48/68) (Table 4). Fifty percent of patients presented with non-surgical/medical complications. CD1 complications accounted for 50% (24/48) of all complications, which were mostly wound-related problems and prolonged ileus. While the Clavien-Dindo Classification only provides information about the worst complication occurring, in our study, 24 patients with a CD1-4 developed multiple complications. Among patients with multiple complications, a detailed morbidity analysis showed that postoperative delirium occurred in 7 patients, and congestive heart failure was observed in 9 cases.

**Table 4:** Postoperative complications according to Clavien-Dindo Classification

	CD1	CD2	CD3a	CD3b	CD4a	CD4b	CD5
**30-day complication**							
Wound problem	9						
Delirium	3						
Ileus	4	2					
Infection (pneumonia, urinary inf, abdominal sepsis)		10			2	1	4
Biliary fistula		1					
Pulmonary embolism					1		
Atrial fibrillation		2					
Blood transfusion		2					
Urinary retention	3						
Congestive heart failure	3						
Heart failure					1		2
Hemoperitoneum				1			
Respiratory failure					2		2
Kidney failure	1						
MOF (multiple organ failure)							1
Pressure ulcers	1						
Complications secondary to progression of an end stage neoplasia							1
**Total**	**24**	**17**	**0**	**1**	**5**	**1**	**10**
**90-day complication**							
Stoma problem	1						
TPN		1					
Respiratory failure (ARDS)					1		
Complications secondary to progression of an end stage neoplasia							2
Pressure ulcers	1						
**Total**	**2**	**1**	**0**	**0**	**1**	**0**	**2**

### Length of stay

The average postoperative length of stay (LOS) was 7 days in the emergency surgery unit and 10.26 days considering the entire hospital admission.

### Functional loss

A severe degree of functional decline was observed in one out of four patients who were alive 30 days after surgery (17/68, 25%). The regression model (Table 5) showed that fTRST≥2, CACI≥6, and age ≥ 85years old had a significant relationship with the loss of independence in ADLs at 30 days. Analysis of the fTRST showed the most accuracy given the area under the curve (AUC) of 71.6 with a sensitivity of 76.5% and specificity of 66.7%. Being ≥85 years old was also a risk factor with a PPV (positive predictive value) of 60%. When fTRST model was adjusted for age as continuous variable, the AUC increased to 80.57%. A statistically significant odds ratio (OR) was obtained for fTRST, CACI≥6, and age ≥ 85 (OR 6.5, 3.67, 8.44 respectively).

**Table 5:** Univariate analysis for ADL≤4

Risk factor	Sensibility	95%CI	Specificity	95% CI	AUC	PPV	95%CI	NPV	95%CI	*p*	Odds ratio	95%CI	*p*
**30 day functional loss** Rate 25% (17/68)													
fTRST≥ 2	76.5%	50.1%, 93.2%	66.7%	52.1%, 79.2%	71.6	43.3%	25.5%, 62.6%	89.5%	75.2%, 97.1%	0.002*	6.50	1.84% 22.98%	0.004*
ASA≥ 4	17.6%	3.8%, 43.4%	94.1%	83.8%, 98.8%	55.9	50%	11.8%, 88.2%	77.4%	65%, 87.1%	0.165	3.43	0.62%, 18.91%	0.157
CACI≥6	64.7%	38.3%, 85.8%	66.7%	52.1%, 79.2%	65.7	39.3%	21.5%, 59.4%	85%	70.2%, 94.3%	0.023*	3.67	1.16%, 11.61%	0.027*
Major surgery	58.8%	32.9%, 81.6%	58.8%	44.2%, 72.4%	58.8	32.3%	16.7%, 51.4%	81.1%	64.8%, 92%	0.206	2.04	0.67%, 6.23%	0.210
Age≥85 years	52.9%	27.8%, 77%	88.2%	76.1%, 95.6%	70.6	60%	32.3%, 83.7%	84.9%	72.4%, 93.3%	<0.001*	8.44	2.35%, 30.28%	0.001*
**90 day functional loss** Rate 21.2% (14/66)													
fTRST≥2	78.6%	49.2%, 95.3%	65.4%	50.9%, 78%	72	37.9%	20.7%, 57.7%	91.9%	78.1%, 98.3%	0.003*	6.93	1.71%, 28.05%	0.007*
ASA≥4	14.3%	1.78%, 42.8%	92.3%	81.5%, 97.9%	53.3	33.3%	4.33%, 77.7%	80%	67.7%, 89.2%	0.469	2.00	0.33%, 12.24%	0.453
CACI≥6	64.3%	35.1%, 87.2%	67.3%	52.9%, 79.7%	65.8	34.6%	17.2%, 55.7%	87.5%	73.2%, 95.8%	0.033*	3.71	1.07%, 12.77%	0.038*
Major surgery	57.1%	28.9%, 82.3%	57.7%	43.2%, 71.3%	57.4	26.7%	12.3%, 45.9%	83.3%	67.2%, 93.6%	0.323	1.82	0.55%, 5.99%	0.326
Age≥85 years	50%	23%, 77%	86.5%	74.2%, 94.4%	68.3	50%	23%, 77%	86.5%	74.2%, 94.4%	0.005*	6.43	1.72%, 23.97%	0.006*

*Statistically significant

Between 31 and 90 days, no patient developed additional functional loss. However, in patients who had functional decline at 30 days, only 3/17 patients recovered by 90 days, and 2 patients died in this period.

The regression model showed a significant relation between 90-day functional loss for fTRST, CACI, and age over 84 years. The most important predictor was the fTRST with an AUC of 72. When fTRST was adjusted per age as continuous variable, the AUC increased again to 77.27.

### Institutionalization

The institutionalization rate was 7.6% (5/66). The median age was 77 years old (range 75–92). All the institutionalized patients had a fTRST≥2. All the patients discharged to a nursing home lived alone or had no caregiver able or available before surgery (a variable also reported in the fTRST). All institutionalized patients developed a surgical complication during the hospital stay; 3 patients had a minor complication (CD1-2) and 2 a major complication (CD 4a—respiratory insufficiency and abdominal sepsis).

## Discussion

The number of older adults admitted every year to surgical departments for an acute condition is rising, and 40–50% of all emergency surgical operations are performed in patients over 65 years of age [21, 22]. In our study, 41.4% of patients admitted to our emergency surgery unit were 70 years old and older. Among this heterogeneous population, frailty is considered the most important risk factor for postoperative adverse events, prolonged length of stay, and functional and cognitive capacity decline [4, 9, 23]. Decision making in an emergency condition can be challenging, and frailty assessment becomes paramount in order to help clinicians identify patients at higher risk for poor outcomes and to discuss strategies and expectations with the patient and relatives. In the emergency setting, a comprehensive frailty assessment cannot usually be performed, not only because it would be too time consuming or because a geriatrician is not always available, but also because ongoing acute conditions incumber a reliable evaluation of the patient. In addition, some domains of the frailty assessment such as walking capacity or mental status evaluation can be altered as a result of the acute condition. A reliable screening tool, feasible before emergency surgery, should have well-defined characteristics: (1) short and fast to perform, (2) requires limited blood tests as many are not available in the emergency setting (i.e., serum albumin level, interleukin), and (3) requires minimal patient collaboration/performance.

In the literature there are few studies on the application of frailty screening tools in the emergency setting. Among those, Joseph et al. [23] applied the modified 50-variable Rockwood Preadmission Frailty Index (50-RPFI) among a geriatric surgical emergency population. This screening tool consists of 50 items and investigates many domains as comorbidities, ADL, IADL, and psychological, functional, and nutritional status, and requires specific blood test. A group of researchers from the University of Arizona validated in two publications [24, 25] a shorter form of this extensive questionnaire selecting the 15 variables more predictive of morbidity and mortality after emergency surgery, named emergency general surgery-specific frailty index (EGSFI). The EGSFI is easier to perform than the 50-RPFI and demonstrates a correlation with complications, mortality, adverse discharge disposition, and 30-day readmission rates [25].

Another screening tool applied in the emergency setting is the 7-point Clinical Frailty Scale (CFS) [26]. It is a simple infographic tool that classifies the patient in seven categories based on a clinician evaluation: very fit, well, well with treated comorbid disease, apparently vulnerable, mildly frail, moderate frail, severely frail. It was applied in different studies in trauma ad emergency surgery [9, 27, 28]. This screening tool seems reliable; however, this classification requires a comprehensive evaluation of comorbidities and several physical and psychological aspects. This clinical judgment requires a thorough knowledge of patient’s past medical history and could be time consuming.

We previously described our experience using the fTRST as frailty screening tool in a population of older patients undergoing urgent abdominal surgery showing that it could accurately predict postoperative morbidity and mortality in this population [10].

In the present study, we investigated the role of the fTRST in predicting loss of independence, as functional recovery and maintenance of the preoperative functional capacity are often the most important outcomes to geriatric patients. Limited previous experiences have been reported in this field. Tan et al. [29] observed a loss of independence 1 year after surgery in 6.9% of the cohort and conclude that frailty (assessed with Modified Fried’s frailty Criteria or with Modified Frailty Index-11) was the stronger predictor of functional decline. Unfortunately, the authors also revealed a major limitation in the application of the Modified Fried’s frailty Criteria as frailty screening tool that they use as it requires to be sufficiently fit to complete the questionnaires, to perform the handgrip tests, and to walk a 15-ft length twice. This overly complex tool is not always feasible in the emergency setting.

In our population, a postoperative functional decline was significantly predicted in patients age more than 85 years, presence of multiple comorbidities (CACI≥6), and if fTRST score was ≥2 both at 30 and 90 days after surgery. The most accurate predictor according to the AUC in our study was found to be fTRST≥2.

Loss of independence has been established to be one of the most important outcomes among aged people because it impacts on patient quality of life, risk of institutionalization, caregiver/family and community burden, and health system financial costs [30, 31]. Functional loss in the geriatric population is often more feared than death; more than 70% of older people would not choose a treatment that causes severe functional disability, even if survival was assured [6] because independence and quality of life are strictly linked.

In our series, after an urgent surgical operation, the inability to perform ADLs among preoperatively independent senior adults was observed in 25% of patients 30 days after surgery. At the 3-month follow-up, we expected a functional recovery. Unfortunately, among the 17 patients who lost independence at 30 days, only 3 patients recovered based on their ADL scores. At the 90-day mark, 2 patients died, 5 were permanently institutionalized, and 7 maintained the acquired decline in the ADL. Among functionally declined patients, at least one postoperative complication occurred within 30 days from surgery in every case. In 88.2% of cases, multiple complications were observed.

In the emergency setting where prehabilitation/preoperative optimization are not possible, the primary strategy to avoid functional decline is a prevention/early management of postoperative complications [5, 32] while adopting the main principles of the enhanced recovery protocols [33]. In our study, 50% of all complications were non-surgical. Many patients reported multiple complications, the most frequent being delirium [7] and congestive heart failure [9]. To avoid loss of independence, a careful management of the postoperative course could help in reducing or mitigating medical complications and potentially impacting early and long-term outcomes. Preventive measures and perhaps establishing a geriatric co-management program have been shown to be effective in managing postoperative delirium, cardiological complications, malnutrition, and promoting physical activity [34]. Shahrokni et al. [35] retrospectively evaluated the effects of geriatricians comanaging a cohort of 1020 patients who underwent elective cancer surgery compared to 872 similar patients who were treated without geriatric comanagement (standard surgical care). The adjusted probability of death within 90 days was less than half in the geriatric comanagement group (4.3% versus 8.9%, 95% CI 2.3–6.9%, *p* <0 .001). Although there were similar complication rates between the two groups, the geriatric comanagement group had greater utilization of supportive care services (e.g., physical therapy, nutrition services) and earlier recognition of geriatric-specific complications which likely contributed to the decreased mortality rate in this group.

Finally, for older patients undergoing surgery, it is of primary importance to identify patient’s social support system (living situation, caregiver able or available) in order to optimize the discharge plan in advance and reduce prolonged hospitalization [34]. In our series, a discharge to a nursing-home facility occurred in 7.6% (5/66) of patients; all of them had a fTRST score≥2.

The main limitations of this study are the small sample size and single-center design. Heterogeneity of the surgical procedures could also represent a limitation; however, once more, we would like to highlight that in the emergency setting functional reserve (and consequently its preoperative evaluation) has a greater role compared to the surgical procedure itself. The follow-up was also limited to 3 months, although the decision to close the monitoring at that time was an attempt to reduce the chance that other causes, independent from the surgery, could influence the patient functional status.

## Conclusion

Among functionally independent older patients, a feared consequence of a surgical intervention is loss of autonomy, becoming a burden for family/caregivers, or becoming institutionalized. Above all in the emergency setting, detection of frailty and careful prevention of postoperative complications are most effective in avoiding functional decline. fTRST is brief and easy to perform and allows identification of vulnerable and frail senior adults to effectively predict functional decline among preoperatively independent patients.

## Data Availability

The dataset generated and analyzed during the current study is available from the corresponding author on reasonable request.

## Abbreviations

ADL: Activities of daily living
fTRST: Flemish version of the Triage Risk Screening Tool
ASA: American Society of Anesthesiology Score
CACI: Charlson Comorbidity Age Adjusted
ESU: Emergency surgery unit
CD: Clavien-Dindo classification
ROC: Receiver operating characteristic curve
AUC: Area under the curve
OR: Odds ratio
AIC: Akaike information criterion
BIC: Bayesian information criterion
50-RPFI: 50-variable Rockwood Preadmission Frailty Index
IADL: Instrumental activities of daily living
CI: Confidence interval
